# Characterization of Metagenome-Assembled Genomes and Carbohydrate-Degrading Genes in the Gut Microbiota of Tibetan Pig

**DOI:** 10.3389/fmicb.2020.595066

**Published:** 2020-12-23

**Authors:** Saisai Zhou, Runbo Luo, Ga Gong, Yifei Wang, Zhuoma Gesang, Kai Wang, Zhuofei Xu, Sizhu Suolang

**Affiliations:** ^1^Department of Animal Science, Tibet Agricultural and Animal Husbandry College, Linzhi, China; ^2^Animal Epidemic Prevention and Control Center of Tibet Autonomous Region, Lasa, China; ^3^Shanghai MasScience Biotechnology Institute, Shanghai, China

**Keywords:** Tibetan pig, gut microbiota, metagenome-assembled genomes, carbohydrate-degrading genes, uncultivated microorganisms, complex carbohydrates

## Abstract

Tibetan pig is an important domestic mammal, providing products of high nutritional value for millions of people living in the Qinghai-Tibet Plateau. The genomes of mammalian gut microbiota encode a large number of carbohydrate-active enzymes, which are essential for the digestion of complex polysaccharides through fermentation. However, the current understanding of microbial degradation of dietary carbohydrates in the Tibetan pig gut is limited. In this study, we produced approximately 145 gigabases of metagenomic sequence data for the fecal samples from 11 Tibetan pigs. *De novo* assembly and binning recovered 322 metagenome-assembled genomes taxonomically assigned to 11 bacterial phyla and two archaeal phyla. Of these genomes, 191 represented the uncultivated microbes derived from novel prokaryotic taxa. Twenty-three genomes were identified as metagenomic biomarkers that were significantly abundant in the gut ecosystem of Tibetan pigs compared to the other low-altitude relatives. Further, over 13,000 carbohydrate-degrading genes were identified, and these genes were more abundant in some of the genomes within the five principal phyla: *Firmicutes*, *Bacteroidetes*, *Spirochaetota*, *Verrucomicrobiota*, and *Fibrobacterota*. Particularly, three genomes representing the uncultivated *Verrucomicrobiota* encode the most abundant degradative enzymes in the fecal microbiota of Tibetan pigs. These findings should substantially increase the phylogenetic diversity of specific taxonomic clades in the microbial tree of life and provide an expanded repertoire of biomass-degrading genes for future application to microbial production of industrial enzymes.

## Introduction

Tibetan pig is a local pig breed mainly distributed in the Qinghai-Tibet Plateau (Yang et al., 2011). It is characterized by stronger adaptability for high-altitude, hypoxia, and cold environments than the lowland pig breeds (Ai et al., 2014; Gan et al., 2019). Like other herbivorous mammals, Tibetan pigs utilize gut microbiota and microbial fermentation in the large intestine to digest plant materials with high fiber content (Wenk, 2001; Yang et al., 2018). As the mammalian genomes lack the enzymes to breakdown plant-derived fibers, degradation of dietary carbohydrates and host-derived glycans mainly depends on the intestinal bacteria and numerous carbohydrate-active enzymes (CAZymes) produced by the primary degraders (Flint et al., 2012). CAZymes can catalyze the degradation of plant cell wall polysaccharides, glycoconjugates, and indigestible oligosaccharides to fermentable monosaccharides (El Kaoutari et al., 2013). The main products of fermentation by anaerobic microbiota in the large intestine are short-chain fatty acids (SCFAs) that play crucial roles in mammalian energy metabolism, gastrointestinal physiology, and immune function (Den Besten et al., 2013; Silva et al., 2020).

Among the known biomass-degrading enzymes, the glycoside hydrolases, polysaccharide lyases, and carbohydrate esterases are the major enzyme activities responsible for the breakdown of complex carbohydrates (El Kaoutari et al., 2013; Abot et al., 2016). Both glycoside hydrolases and polysaccharide lyases can cleave glycosidic bonds of various carbohydrate substrates, for instance, cellulose, xylan, pectin, hyaluronan, and heparin (El Kaoutari et al., 2013). Glycoside hydrolases are the most abundant enzymes with broad substrate specificity in the gut microbiota of mammals (Lee et al., 2014). Carbohydrate esterases are a class of esterases involved in the degradation of acetylated plant hemicelluloses (Biely, 2012). The diversity of carbohydrate-degrading enzymes has been studied in many host-related habitats (e.g., digestive tract and rumen) (El Kaoutari et al., 2013; Lee et al., 2014; Stewart et al., 2019). Identification of degradative enzymes is of great significance for industrial applications, such as biocatalysts, biorefining, textiles, and food-processing (Kirk et al., 2002).

During the last decade, high-throughput sequencing technology has extensively promoted microbial community studies to explore taxonomic diversity and to discover novel carbohydrate-active proteins in the microbiome. A reference gene catalog of the pig gut microbiome has been established using large-scale metagenome shotgun sequencing (Xiao et al., 2016a). The clustering analysis at the gene level has further pointed out that Tibetan pigs with high-altitude adaptation form a unique group that is distinct from the other breeds, revealing the impact of host genetics on the composition of the gut microbiota. Based on 16S rRNA amplicon sequencing, a recent study on the pig cecal microbiota has detected differentially abundant taxa between the Tibetan and PIC (lean-type) pigs, including significantly abundant *Bacteroidetes* and *Spirochaetota* involved in cellulose degradation in the Tibetan pigs, and more abundant *Proteobacteria* dominated by the genera *Campylobacter* and *Helicobacter* in the PIC pigs (Yang et al., 2018). Like the other common pig breeds (i.e., Hampshire, Landrace, Duroc, and Yorkshire) (Xiao et al., 2017; Yang et al., 2018), the gut microbiome of Tibetan pigs is dominated by *Firmicutes* and *Bacteroidetes*, which are also the two major phyla in the gut microbiota of human (Segata et al., 2012) and rumen microbiota of cattle (Stewart et al., 2018b). On the other hand, metagenome sequencing data can be used to recover uncultivated microbial genomes from environments and host-related niches. Many of the recovered genomes represent the newly identified species beneficial for the discovery of enzymes with industrial potential (Almeida et al., 2019). For instance, a large metagenome sequencing study by Stewart et al. has reconstructed 4,941 bacterial and archaeal genomes, most of which represent newly uncultured rumen species. These genomic assemblies enable the further identification of a mass of enzymes participating in the digestion of plant materials that dominate the cow diet (Stewart et al., 2019).

To uncover the previously unknown microbial species with the potential to degrade complex carbohydrates in the gut, we performed shotgun metagenomic sequencing of fecal samples collected from Tibetan pigs. Metagenomic assembly and binning reconstructed 322 near-complete and draft prokaryotic genomes. The abundance profiles of the genomes were investigated to infer putative metagenomic biomarkers in the microbiome of Tibetan pigs. Furthermore, we characterized a substantial number of carbohydrate-degrading enzymes and polysaccharide utilization loci in the genomes derived from the uncultivated species belonging to certain clades within the principal phyla.

## Materials and Methods

### Sample Collection

The samples were collected from eleven healthy adult Tibetan pigs in two farms in Tibet, China. The details on the collection dates and geographical locations were summarized in Supplementary Table 1. Fresh fecal samples were collected and stored using the fecal collection tubes in the Longseegen Stool Storage Kit (Longsee Biomedical Corporation, Guangzhou, China) according to the manufacturer’s protocol.

### Shotgun Metagenomic Sequencing

The experiments of Illumina sequencing were conducted at Novogene, China. Briefly, total genomic DNA was extracted using the QIAamp DNA Stool Mini Kit (Qiagen, Hilden, Germany). DNA integrity and purity were assessed using Agarose Gel Electrophoresis and Nanodrop ND1000 (Thermo Fisher Scientific, United States). ∼ 1 μg DNA was sheared into ∼350 bp insertion fragments by the Covaris M220 instrument (Covaris, MA, United States). Sequencing libraries were then constructed using a TruSeq DNA library kit (Illumina Inc., CA, United States). Sequencing of paired-end 150-bp reads was carried out using an Illumina NovaSeq 6000 sequencer, generating > 10 Gb of raw reads per sample. Quality control of raw reads was performed using Trimmomatic v0.39 with the following parameter criteria: -threads 16 -phred33 LEADING:20 TRAILING:20 SLIDINGWINDOW:4:20 MINLEN:40 AVGQUAL:20 (Bolger et al., 2014). The host contaminations were then removed by mapping all trimmed reads to the reference genome of *Sus scrofa* (NCBI RefSeq assembly: GCF_000003025) using BMTagger (Rotmistrovsky and Agarwala, 2011). The quality report of metagenomic reads per sample was generated using FastQC v0.11.8 (Brown et al., 2017). The number of paired reads after quality trimming and host DNA removal, respectively, is shown in Supplementary Figure 1.

### Metagenomic Assembly and Binning

To reconstruct microbial genomes from metagenomic data, we adopted an integrative workflow framework for individual sample assembly, binning, de-replication, and removal of contig contamination as described previously (Olm et al., 2017; Stewart et al., 2019; Uritskiy and Diruggiero, 2019). Metagenomic assembly and contig binning were carried out using the pipeline MetaWRAP v1.2.2 (Uritskiy et al., 2018). Briefly, metagenomic reads per sample were corrected by BayesHammer and then assembled using metaSPAdes v3.13.0 (Nurk et al., 2017). The *de novo* assembly was performed using the default parameters except for -t 36 -m 200 -l 1000. The number of the assembled contigs (≥1 kb) across the metagenomes is 137,805 (representing 412 Mb) to 239,091 (670 Mb). Genome binning was carried out for all the contigs longer than 2 kb using two computational approaches MetaBat v2.12.1 (Kang et al., 2019) and MaxBin 2.2.6 (Wu et al., 2015), respectively. A further refinement on both sets of bins was performed to produce a combined and robust bin set using the MetaWRAP Bin_refinement module with the parameters -m 150 -t 18 -c 50 -x 10. All the resulting genome bins (*n* = 1,095) from all samples were then pooled together and de-replicated using the dRep package v2.3.2 with the following parameters: -comp 80 -con 10 -strW 1 -p 36 –S_algorithm ANImf –length 100000 –checkM_method lineage_wf (Olm et al., 2017). The putative contig contamination per genome was detected and removed by using MAGpurify v2.1.2 (Nayfach et al., 2019). Genome quality was estimated using CheckM v1.0.12 to calculate genome completeness and contamination (Parks et al., 2015). All the genomes with completeness ≥ 80% and contamination ≤ 10% were retained for the following analyses. To assess the genomic coverage across the sequenced metagenomes, read mapping to the genomes was performed for each sample using Minimap v2.17 (Li, 2018). CoverM v0.5^1^ was then used to calculate relative abundance and coverage percentages of each genome with the option -m relative_abundance covered_fraction.

### Taxonomic and Phylogenetic Analysis of MAGs

Taxonomic lineages of the reconstructed MAGs were inferred using the recently developed database GTDB release v95 (Parks et al., 2018) and the relevant toolkit GTDB-Tk v1.2.0 (Chaumeil et al., 2019). GTDB implemented a new microbial taxonomy based on domain-specific reference trees, which can be used for robust classification of the query genomes by GTDB-tk. Briefly, protein-coding genes were called by Prodigal 2.6.3 (Hyatt et al., 2010), and marker genes per genome were identified with a set of 120 bacterial and 122 archaeal genes using HMMER v3.1b2 (Finn et al., 2011). The selected marker genes were aligned and concatenated into a single alignment for placement of genomes onto the domain-specific reference trees using pplacer v1.1 (Matsen et al., 2010). Taxonomic assignment to a query genome was then determined according to a combination of its placement in the GTDB reference tree, relative evolutionary divergence, and Average Nucleotide Identity (ANI) to reference genomes (Chaumeil et al., 2019).

The phylogenomic analysis for MAGs was performed with the package PhyloPhlAn v1.0 (Segata et al., 2013), supporting large phylogenetic reconstructions of hundreds of microbial genomes. Briefly, according to the PhyloPhlAn non-redundant database comprising 400 most ubiquitous proteins in prokaryotic genomes, the homologs encoded in the genomes were searched using USEARCH v5.2.32 (Edgar, 2010). Multiple sequence alignments of the selected protein markers were carried out using MUSCLE v3.8.1551 (Edgar, 2004) and were then concatenated for whole-genome phylogenetic reconstruction using FastTree v2.1 (Price et al., 2010). The resulting tree was midpoint rooted using FigTree v1.4.3^2^. Phylogenetic structure and taxonomic information of the genomes were integrated and visualized by using GraPhlAn (Asnicar et al., 2015).

### Annotation of Carbohydrate-Active Function

For each genome, the genes encoding carbohydrate-active enzymes (CAZymes) were predicted and annotated using dbCAN2 (Zhang et al., 2018), which curated a set of HMMs corresponding to the CAZyme families created by the CAZy database (Lombard et al., 2014). HMMER v3.1b2 (Finn et al., 2011) was used to search the HMMs of dbCAN2, and the result was filtered with the thresholds below: *E*-value < 1e-15 and coverage > 0.35. Polysaccharide utilization loci (PULs) in microbial genomes were predicted using PULpy that implemented a pipeline to identify CAZymes co-localized with tandem gene pairs *susCD* (Stewart et al., 2018a). Default parameters in the PULpy pipeline were adopted.

### Statistical Analysis

Metagenomic biomarkers were inferred based on the abundance estimates of MAGs using the algorithm of linear discriminant analysis (LDA) effect size (LEfSe) (Segata et al., 2011). Differentially abundant features were determined by using the Kruskal–Wallis (KW) sum-rank test and the estimated effect size. The minimum LDA score was set to 2.5, and the maximum *p*-value for the KW test was set to 0.001.

### Data Availability

The raw reads of metagenome sequencing have been submitted to the NCBI SRA database under the accession PRJNA647157.

## Results

### General Features of 322 Microbial Genomes Assembled From the Fecal Microbiome

In the present study, metagenome sequencing on the fecal samples from 11 animals yielded about 492 million reads and 145 gigabases in total. After a continuous assembly and dereplication analysis workflow, a set of unique strain-level genome bins (<99% ANI) was obtained. Here, we presented 322 metagenome-assembled genomes (MAGs) with completeness greater than 80% and contamination less than 10%. These reconstructed prokaryotic genomes were described as a gut mini-microbiome in Tibetan pigs.

The assembly quality statistics of the 322 genomes are summarized in Supplementary Table 2. The total sequence length of all MAGs was 615 megabases (Mb). The genome sizes of individual MAGs were ranged from 727 kilobases (kb) to 4.3 Mb, with N50 values ranging from 5.5 to 212.3 kb. The average GC% content of the genomes had a wide range from 25 to 70%. Among these genome bins, 121 were estimated to be near-complete genomes with completeness ≥ 90% and contamination ≤ 5% (Parks et al., 2017). Furthermore, 28 genomes were >95% complete. Particularly, MAG001 belonging to the archaeal genus *Methanobrevibacter* was found to have 100% completeness and 0% contamination with 63 contigs (∼1.52 Mb in size).

### Taxonomic Annotation of the Genomes

**FIGURE 1:**
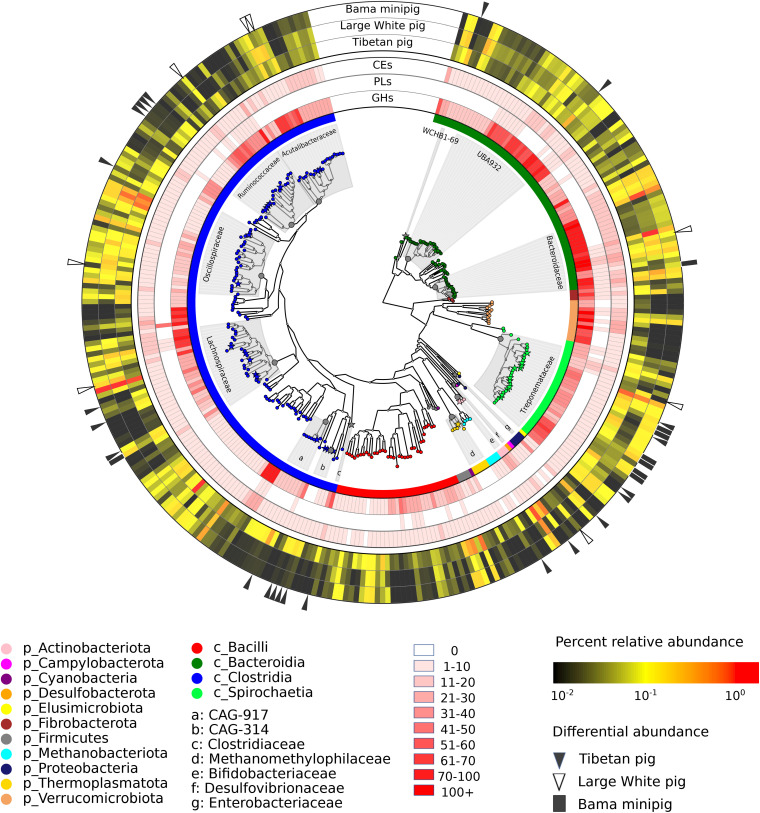
The characterizations of 322 metagenome-assembled genomes and their carbohydrate degrading enzymes from the fecal microbial community of Tibetan pigs. The internal tree denotes whole-genome phylogenetic relationships among the recovered genomes. The outer rings range from 1 (the innermost ring) to 8 (the outermost ring). The innermost ring and the enlarged leaf nodes are color-coded by the taxonomic distribution of genomes whose organismal identifiers at the phylum or class level are displayed under the circular map. The next three rings show the distribution of carbohydrate-degrading enzymes. The red gradients are in proportion to the numbers of carbohydrate-degrading enzymes encoded in the individual genomes, and the corresponding statistics are listed in Supplementary Table 7. The 5th to 7th rings show the relative abundances of individual genomes in the gut microbial communities of Tibetan pigs, Large White pigs, and Bama minipigs. In these rings, mean values of percent relative abundances are color-coded. The related abundance data of the genomes across the samples are summarized in Supplementary Table 4. The outermost ring shows differentially abundant genomes detected to be metagenomic biomarkers: black triangles stand for the genomes most abundant in the Tibetan pigs, white triangles for the Large White pigs, and black rectangles for the Bama minipigs. In the internal tree, the star leaf nodes denote differential genomes, and their family identifiers are labeled in the gray sectors.

Next, we investigated taxonomic clades of the individual genomes using the Genome Taxonomy Database (GTDB) that enables objective classification for bacterial and archaeal genomes assembled from the metagenomic datasets (Parks et al., 2018; Chaumeil et al., 2019). A whole-genome phylogenetic tree of the 322 genomes together with some public genomes from closely related species is displayed in Figure 1. It was apparent that the tree was dominated by the bacterial genomes derived from three phyla: *Firmicutes* (188), *Bacteroidetes* (69), and *Spirochaetota* (30), accounting for almost 90% of all MAGs (Supplementary Table 2). Moreover, most genomes within *Firmicutes* were affiliated to the two largest clades *Clostridia* (148) and *Bacilli* (36). The other bacterial genomes were affiliated to eight phyla, including *Verrucomicrobiota* (12), *Proteobacteria* (4), *Actinobacteria* (3), *Fibrobacterota* (3), *Campylobacterota* (1), *Cyanobacteria* (1), *Desulfobacterota* (1), and *Elusimicrobiota* (1). At the family level, the genomes belonging to *Treponemataceae* (28) under the phylum *Spirochaetota* were the top abundant, and all 28 genomes were assigned to a single genus *Treponema*. Four families within *Firmicutes* constituted the prevalent clostridial populations, including *Lachnospiraceae* (26), *Oscillospiraceae* (26), *Acutalibacteraceae* (20), and *Ruminococcaceae* (19). Both UBA932 (26) and *Bacteroidaceae* (18) were the predominant families within *Bacteroidales*. Also, we assembled nine archaeal genomes, which were affiliated to two families: five genomes belonging to *Methanomethylophilaceae* within the *Thermoplasmatota* phylum and four belonging to *Methanobacteriaceae* within *Methanobacteriota*, respectively.

Overall, all 322 genomes can be taxonomically assigned to at least the order level by GTDB (see details in Supplementary Table 2). At the lower taxonomic ranks, the numbers of the genomes classified at the family, genus, and species levels were 321, 295, and 131, respectively. Based on the ANI analysis, a total of 191 genomes that share < 95% ANI with the reference genomes of GTDB may represent uncultivated bacteria and archaea from novel taxa. Notably, only 38 genomes had validly published taxon names at the species rank, and the remaining were unclassified or assigned the GTDB-proposed placeholder names for uncultivated species (Parks et al., 2020). Some of the genomes representing the well-studied species may be involved in the breakdown of non-digestible dietary polysaccharides and fermentation to produce SCFAs (butyrate, acetate, and propionate), which are vital metabolites responsible for mammalian energy metabolism and gut homeostasis (Den Besten et al., 2013; Rivière et al., 2016). For example, MAG079 represented the genome derived from the uncultivated strain of *Ruminococcus flavefaciens*, which is an important cellulolytic bacterium capable of digesting cellulose and hemicellulose plant cell walls (Fontes and Gilbert, 2010). Two MAGs (#005 and #231) represented the genomes from the *Bifidobacterium* species *B. pseudolongum* and *B. thermacidophilum*, both of which are butyrate-producing bacteria involved in the maintenance of the gut barrier functions (Rivière et al., 2016). And MAG022 represented the genome from the uncultivated strain of *Coprococcus catus*, which can produce both butyrate and propionate (Ríos-Covián et al., 2016).

### Analysis of Genome Coverage and Biomarkers

To investigate the community composition of uncultured microorganisms, genome coverage and relative abundances were estimated by recruiting the reads to the genome sequences of MAGs. As a result, we found on average, ∼38% of the reads were mapped to the recovered genomes across the metagenomes of Tibetan pigs. Using a cutoff of >80% coverage, 126 MAGs were present in at least five animals, and seven MAGs were present in all animals (Supplementary Table 3). Notably, seven highly covered MAGs represented the uncultivated clostridial strains from six uncharacterized genera (CAG-83, CAG-170, CAG-177, UBA2868, UBA738, and ER4) defined by GTDB (Supplementary Table 2).

Furthermore, to explore whether some microbial species are significantly abundant in the gut microbiota of Tibetan pigs, we compared genomic abundances with the collected metagenomic data sets of the commercial porcine breed Large White and the native Chinese Bama minipig (Xiao et al., 2016b). Based on the default cutoff of the LEfSe analysis (Segata et al., 2011), 153 MAGs were estimated to be discriminative features among three pig breeds (Supplementary Table 4). Using more strict criteria (LDA score > 2.5 and KW test *p*-value < 0.001), 39 MAGs were inferred to be putative genomic biomarkers that were significantly abundant in at least one of the three pig breeds: 23 in Tibetan pigs, 8 in Large White pigs, and 8 in Bama minipigs (Figure 1). The family level taxonomic assignments of these differential genomes are shown in Figure 1. The significantly abundant genomes detected in the microbial community of Tibetan pig were affiliated to the following orders: *Christensenellales* (5), *Oscillospirales* (5), *Treponematales* (4), *Lachnospirales* (3), *Bacteroidales* (2), *Clostridiales* (1), *Actinomycetales* (1), *Enterobacterales* (1), and *Methanomassiliicoccales* (1).

### The Repertoire of Carbohydrate-Degrading Genes in the Mini-Microbiome

**FIGURE 2:**
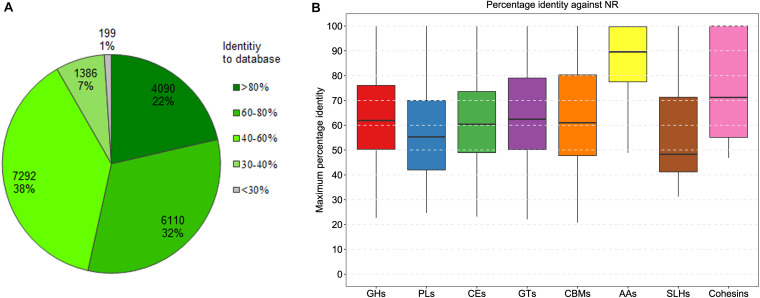
Distribution of the percentage sequence identity between the predicted CAZymes and the best hits from NCBI NR. **(A)** The frequency distribution of the best BLAST hits fell into percentage identity intervals for all the predicted CAZymes. **(B)** The distribution for eight classes of CAZymes. GHs, glycoside hydrolases; PL, polysaccharide lyases; CEs, carbohydrate esterases; GTs, glycosyl transferases; CBMs carbohydrate-binding modules; AAs, auxiliary activities enzymes; SLHs, S-layer homology domains; Cohesins, cohesin domains.

To understand the digestibility of complex polysaccharides by the Tibetan pig gut microbiota, we analyzed carbohydrate-active enzymes encoded in the mini-microbiome. Here, a total of 567,316 protein-coding genes were predicted in the 322 genomes. Using dbCAN2 (Zhang et al., 2018), 19,077 genes coding for the putative CAZymes were identified (Supplementary Table 5). Among these genes, 1,371 code for at least two CAZy family domains per gene. To explore the novelty of the predicted CAZymes, we performed sequence similarity searching against the NCBI NR database. The frequency distribution of the percentage identity values retrieved from the best hits is shown in Figure 2A. Of the 19,077 CAZymes, 4,090 (22%) share more than 80% amino-acid sequence identity with the database entries, and 10,200 (53%) share more than 60% identity with the database entries. It can be assumed that nearly half of the CAZymes with >60% amino acid identities are conserved enzymes that have very comparable catalytic properties. Furthermore, 7,292 proteins with 40–60% identities are likely to be the conserved enzymes with different substrate/linkage specificity. The remaining 1,386 enzymes with 30–40% identities may act on the same substrates, but products and specificity unknown.

According to the enzymatic classification defined by CAZy (Lombard et al., 2014), the repertoire of the genes encompassing carbohydrate-active domains was divided into eight classes, including 10,623 glycoside hydrolases, 322 polysaccharide lyases, 2,543 carbohydrate esterases, 5,761 glycosyl transferases, 1,291 carbohydrate-binding modules, 65 auxiliary activities enzymes, 41 S-layer homology domains, and five cohesin domains. Among these, the first three classes (i.e., glycoside hydrolases, polysaccharide lyases, and carbohydrate esterases) associated with carbohydrate-degrading enzyme activities were the most abundant, accounting for about 65% of all the CAZymes-encoding genes. The distribution of the maximum percentage identity of the best hit for each CAZy enzyme class is shown in Figure 2B. Among all the CAZyme-related genes, the ones encoding AAs are highly conserved, with a median amino-acid identity around 90%; whereas, the genes encoding S-layer homology domains are more divergent, with a median identity around 49%. The other five classes (i.e., glycoside hydrolases, polysaccharide lyases, carbohydrate esterases, glycosyl transferases, and carbohydrate-binding modules) share similar identity medians between 55 and 62%.

Next, the distribution of genes encoding carbohydrate-degrading enzymes in the individual genomes is displayed in Figure 1. The numbers of the genes affiliated to different phyla are summarized in Table 1. On average, each member of the phylum *Verrucomicrobiota* encodes the most carbohydrate-degrading enzymes (98), followed by *Fibrobacterota* (69), *Bacteroidetes* (65), *Spirochaetota* (42). The *Firmicutes* contains the most genomes (188 out of the 322 genomes) but a moderately mean number (31) of glycoside hydrolases, polysaccharide lyases, and carbohydrate esterases per genome. On the other hand, the distribution of carbohydrate-degrading genes at the family level is summarized in Supplementary Table 6. The bacteria from both families *Bacteroidaceae* and *Fibrobacteraceae*, which have been recognized as fibrolytic gut bacteria (Abdul Rahman et al., 2015; Xu et al., 2017), possessed 85 and 54 glycoside hydrolases per genome, respectively. It was apparent that some uncharacterized bacterial families also had high numbers of glycoside hydrolases, such as UBA1829 (144 GHs per genome), UBA1067 (142), UBA3663 (91), CAG-74 (90), and UMGS1810 (76). The families mentioned above were affiliated to the phyla *Verrucomicrobiota* (UBA1829 and UBA1067), *Bacteroidetes* (UBA3663), *Firmicutes* (CAG-74 and UMGS1810), respectively.

**TABLE 1:** Summary of the genes encoding carbohydrate-degrading enzymes affiliated to different phyla in the mini-microbiome.

**Phylum**	**Genomes**	**GH genes**		**PL genes**		**CE genes**		**Total genes**	
		**No.**	**Mean**	**No.**	**Mean**	**No.**	**Mean**	**No.**	**Mean**
*Actinobacteriota*	3	70	23.3	0	0	22	7.3	92	30.7
*Bacteroidetes*	69	3590	52.0	124	1.8	738	10.7	4452	64.5
*Campylobacterota*	1	1	1.0	0	0	0	0	1	1.0
*Cyanobacteria*	1	13	13.0	0	0	2	2.0	15	15.0
*Desulfobacterota*	1	7	7.0	0	0	1	1.0	8	8.0
*Elusimicrobiota*	1	9	9.0	0	0	3	3.0	12	12.0
*Fibrobacterota*	3	162	54.0	17	5.7	29	9.7	208	69.3
*Firmicutes*	188	4474	23.8	119	0.6	1287	6.8	5880	31.3
*Methanobacteriota*	4	6	1.5	0	0	3	0.8	9	2.3
*Proteobacteria*	4	54	13.5	0	0	16	4.0	70	17.5
*Spirochaetota*	30	1044	34.8	34	1.1	180	6.0	1258	41.9
*Thermoplasmatota*	5	0	0	0	0	5	1.0	5	1.0
*Verrucomicrobiota*	12	978	81.5	19	1.6	174	14.5	1171	97.6

To further explore uncultivated bacteria with the potential to degrade complex polysaccharides in the diet for Tibetan pigs, we created a plot to highlight the genomes according to the numbers of both carbohydrate-degrading enzymes and the related CAZy families (Supplementary Figure 2). Interestingly, high numbers of the degradative enzymes were observed in the three *Verrucomicrobiota* genomes; both MAG202 (185 GHs, 7 PLs, ad 27 CEs) and MAG267 (179 GHs, 5 PLs, ad 20 CEs) were the uncultivated *Kiritimatiellae* species, and MAG200 (144 GHs, 5 PLs, ad 27 CEs) was an uncultivated *Victivallales* species. The density of genes encoding glycoside hydrolases in the three genomes mentioned above was 54.8, 50.0, and 40.2, respectively (Supplementary Table 7). Numerous carbohydrate-degrading enzymes and highly diversified CAZy families were also found in certain genomes belonging to the *Bacteroidetes*, *Firmicutes*, *Fibrobacterota*, and *Spirochaetota* (Supplementary Figure 2). For instance, two genomes (MAG #160 and #137), which are both the members of *Prevotella* within *Bacteroidaceae*, encode 154 and 146 degradative enzymes, respectively. The genes encoding polysaccharide lyases were present in only 63 of the 322 genomes, and the total number (313) of genes was the fewest among three classes of carbohydrate-degrading enzymes (Figure 1). Notably, MAG052 representing the genome from the uncultivated *Clostridia* had significantly abundant polysaccharide lyases (50) assigned to six families (i.e., PL35, PL12, PL21, PL27, PL33, and PL8). Among all genomes, the second-largest number of polysaccharide lyases was 12 found in the MAG114, which represented an unclassified species from the genus *Treponema*. In addition, the genes encoding cellulases (GH5, GH8, GH9, GH44, GH45) were frequently observed in the three *Fibrobacterota* MAGs (#218/#236/#011), and the corresponding gene densities of glycoside hydrolases were 21.5, 19.8, and 18.9, respectively. Consistently, all three genomes belonged to *Fibrobacter*, a genus consisting of highly cellulolytic bacteria in herbivore guts (Abdul Rahman et al., 2015).

### Diversification of PULs

A polysaccharide utilization locus is a genomic region that encodes the necessary proteins involved in the biological processes from the initial binding of a particular polysaccharide at the cell surface to the final saccharification of the cleavage oligosaccharides to monosaccharides in the periplasmic space (Terrapon et al., 2015). PULs are usually present in the genomes derived from many organisms of *Bacteroidetes*, which are characterized by tandem *susC*/*susD* pairs and adjacent genes encoding regulators, binding proteins, and the CAZymes specific to one or multiple substrates (Terrapon et al., 2015). Here, we scanned the presence of polysaccharide utilization gene clusters in the 322 genomes. As a result, 452 putative PULs were identified in 59 genomes, all of which represented the members of *Bacteroidetes* (Supplementary Table 8). Of these genomes, 54 encode more than one polysaccharide utilization locus. The largest number of PULs per genome was 32 presenting in MAG105, which represented an unclassified species from the genus RC9 under the *Bacteroidales* by GTDB. The second-largest number was 25 presenting in MAG160 derived from an unclassified species within *Prevotella*. The members of *Prevotella* have been often reported for their ability to utilize multiple polysaccharides as substrates, like *P. multisaccarivorax* (Sakamoto et al., 2005) and *P. copri* (Fehlner-Peach et al., 2019).

Besides, a total of 1,894 protein-coding genes were encompassed by all detected PULs. The patterns of the polysaccharide utilization gene clusters were highly diversified in the Tibetan pig gut microbiome. The most frequently observed pattern was a single *susC*/*susD* pair, and such organization was found in 226 of all the gene clusters (Supplementary Table 8). This simple genetic organization has also been found to be the most in the PULs encoded in the recovered genomes from the rumen microbiome (Stewart et al., 2018b). The other organizations of the polysaccharide utilization gene clusters exhibited great diversity, which was characterized by various combinations of distinct CAZy families and the proteins of unknown function. The most common degradative enzymes encoded in the polysaccharide utilization gene clusters were glucoamylases (GH97), α-amylases (GH13), endoglucanases (GH5), β-xylosidases (GH43), β-galactosidases (GH2), and β-glucosidases (GH3) involved in the breakdown of starch, cellulose, and oligosaccharides. The genes encoding enzymes (GH10, GH16, GH26, GH28, and GH32) to hydrolyze hemicellulosic polysaccharides were frequently observed in the polysaccharide utilization loci.

## Discussion

As one of the high-altitude domestic animals, Tibetan pigs have evolved distinctive capabilities for the adaptation to cold temperatures, hypoxia, and poor feeding conditions (Ai et al., 2014). They provide meat products of high nutritional value for millions of people living in the Qinghai-Tibet Plateau (Gan et al., 2019). In recent years, numerous studies have pointed out that mammalian intestinal microbiotas have important beneficial effects on host energy metabolism and nutrient absorption (Flint et al., 2012; Krajmalnik-Brown et al., 2012; Den Besten et al., 2013). Particularly, the gut microorganisms enable providing a large panel of enzymes involved in the degradation of complex carbohydrates, including plant cell wall polysaccharides, storage carbohydrates, and host glycans (El Kaoutari et al., 2013; Ilmberger et al., 2014; Milani et al., 2017; Wang et al., 2019). To understand the microbial degradation of dietary carbohydrates in the gut ecosystem of Tibetan pigs, draft microbial genomes were reconstructed through metagenomic sequencing and binning in this study. Among the uncultivated microbial populations represented by the recovered genomes, some were found to be putative organismal indicators specific to the gut ecosystem of Tibetan pig. Further, the diversity of carbohydrate-degrading enzymes was characterized in the genomes from certain clades within five principal phyla.

Recovery of the genomes using culture-independent approaches has recently become a commonly used practice for species discovery and characterization (Parks et al., 2017; Almeida et al., 2019; Wang et al., 2019). In this study, we assembled 313 bacterial genomes and nine archaeal genomes, most of which represented as-yet uncultivated species inhabiting the animal gut ecosystem. Particularly, 23 genomes were identified as highly abundant and putative biomarkers in the gut microbiome of Tibetan pigs comparing to the other low-altitude relatives, Large White and Bama minipigs. The microbial species represented by the genomic indicators may play important physiological and biochemical roles in the gut environment of Tibetan pigs. Among all the MAGs, the ones (14) derived from the *Clostridia* class were the most. They represented the novel bacterial taxa mainly distributed in the two well-studied families *Ruminococcaceae* (4) and *Lachnospiraceae* (3), as well as in an uncharacterized family CAG-314 (4) belonging to a new order *Christensenellales* (Parks et al., 2018). Moreover, these clostridial genomes were found to be present in most animals (Supplementary Table 3), indicating their prevalence and high abundance may play active roles in the lifestyle of Tibetan pigs. Many commensal species of *Clostridia*, a large group of obligate anaerobic and highly polyphyletic bacteria, are considered responsible for the maintenance of gut homeostasis (Lopetuso et al., 2013). The high diversity of uncultivated and unclassified genomes affiliated to *Clostridia* has also been reported in the human gut microbiome (Almeida et al., 2019) and the bovine rumen microbiome (Stewart et al., 2019). Notably, many members of both families, *Ruminococcaceae* and *Lachnospiraceae*, are the major butyrate-producing species that have the metabolic capability to degrade and utilize plant-derived fibers as nutrients (Flint et al., 2012; Milani et al., 2017; Xu et al., 2017). Although the uncultivated CAG-314 species lack sufficient understanding of the bacterial physiological and biochemical properties, the other relative *Christensenellaceae* within the order *Christensenellales* has been described as an important player in human health, which is negatively correlated to host body mass index (Waters and Ley, 2019). Besides, four spirochetal genomes affiliated to uncharacterized *Treponema* species were detected as biomarkers in the Tibetan pig gut microbiome. Six out of eight significantly abundant genomes found in the Bama minipig gut microbiome were also derived from *T. porcinum* and the uncultured species belonging to *Treponema* (Figure 1). Due to the unculturability of some *Treponema* species, the previous metataxonomic studies have utilized 16S rRNA gene amplicon sequencing to reveal the diversity of treponemes (Hallmaier-Wacker et al., 2019). For instance, Angelakis et al. (2019) have reported the gut microbiota of traditional rural individuals is enriched with *Treponema* species, which are absent from urban individuals. Compared to 16S amplicon sequencing, shotgun metagenomic sequencing provides greater genomic coverage that is beneficial for the inference of specific metabolic capabilities of the newly identified microbial taxa in this study.

As is well known, the genomes of individual gut bacteria often encode hundreds of enzymes that play metabolic roles in the degradation of indigestible dietary polysaccharides in mammals (El Kaoutari et al., 2013; Ilmberger et al., 2014). The increasing availability of high-quality microbial genomes assembled from metagenomic sequencing data enables a better understanding of the carbohydrate-active functions of uncultivated and uncharacterized microbes (Luis and Martens, 2018; Stewart et al., 2018b). In this study, we also identified new genomes from the bacterial populations that have been rarely reported about their abilities involved in the metabolism of complex carbohydrates. Strikingly, we identified three *Verrucomicrobiota* genomes representing the uncultivated bacteria with high catalytic potential for the degradation of multiple substrates. Of these genomes, MAG202 belonging to *Kiritimatiellae* possessed the most abundant enzymes assigned to highly diversified CAZy families for the deconstruction of complex polysaccharides, including 47 families of glycoside hydrolases, six families of polysaccharide lyases, and 12 families of carbohydrate esterases. Enzymes frequently present in the MAG202 comprise β-galactosidase, β-xylosidase, glucosidase, xyloglucosyltransferase, cellulase, α-L-fucosidase, rhamnogalacturonan endolyase, acetyl xylan esterase, and arylesterase. The activities of these enzymes should enable a broad substrate specificity for this bacterium. *Kiritimatiella glycovorans*, a representative species also from *Kiritimatiellae*, is known to be obligately anaerobic and saccharolytic bacteria involved in the utilization of multiple substrates (e.g., cellobiose, galactose, fructose, xylose) (Spring et al., 2016). Notably, another *Verrucomicrobiota* genome (MAG200) encodes high numbers of enzymes degrading O-linked and N-linked animal glycans, which were well represented by the following families: GH163, GH109, GH20, GH130, and GH123 (El Kaoutari et al., 2013; Wang et al., 2019).

*Firmicutes* and *Bacteroidetes* dominated the phylogenetic tree of the gut microbial populations in Tibetan pigs. The two bacterial populations are also prevalent in the datasets of draft genomes recovered from the microbial communities of piglet feces (Wang et al., 2019) and the bovine rumen (Stewart et al., 2019). Meanwhile, the genomes from both phyla encode a substantial number of carbohydrate-degrading enzymes and highly diversified CAZy families involved in the utilization of diet- and host-derived carbohydrates (Table 1). For instance, MAG156 harbored 51 glycoside hydrolase families (116 genes), which was the most abundant among 322 genomes (Supplementary Table 7). MAG156 represented the genome of an uncultured species from a new genus UBA4372 under *Bacteroidaceae*. The enzymes frequently observed in the MAG156 include β-xylosidase (GH43), β-galactosidase (GH2), α-amylase (GH13), glucoamylase (GH97), α-L-rhamnosidase (GH78), endoglucanases (GH5), α-L-rhamnosidase (GH106), acetyl xylan esterase (CE1), and arylesterase (CE10) (Supplementary Table 8). The catalytic activities of the above enzymes enable the degradation of plant biomass, including cellulose (GH5), hemicellulose (GH78 and CE1), oligosaccharides (GH43 and GH2), starch (GH13 and GH97), and pectin (GH106) (Pope et al., 2010; Konietzny et al., 2014; Gharechahi and Salekdeh, 2018). Additionally, the clostridial genome (MAG052) encodes many polysaccharide lyases, of which 39 were assigned to the most abundant family PL35 acting on chondroitin (Helbert et al., 2019). Moreover, the enzymes associated with the breakdown of O-linked and N-linked host glycans were encoded in the MAG052, including GH20, GH109, GH123, GH125, CE9, and CE14 (Wang et al., 2019). These results imply that the degradation of complex plant polysaccharides and host glycans in Tibetan pigs may be associated with a lot of enzymatic synergy by the numerous uncultivated microorganisms.

In summary, 322 nearly complete and draft genomes were reconstructed from the fecal microbiome of Tibetan pigs, representing a broad range of microbial populations from 11 bacterial phyla and two archaeal phyla. More than half of the genomes may represent uncultivated microorganisms from novel prokaryotic taxa, which should substantially increase the phylogenetic diversity of specific clades in the microbial tree of life. Further, we identified over 13,000 carbohydrate-degrading enzymes and 452 polysaccharide utilization loci. Remarkably, three genomes representing the uncultivated bacteria within *Verrucomicrobiota* encode the most abundant degradative enzymes in the Tibetan pig gut microbiome. These findings provide a useful genetic repertoire for future research into the uncharacterized microorganisms and microbial enzymes involved in the breakdown of complex carbohydrates in the gut.

## Data Availability Statement

The datasets presented in this study can be found in online repositories. The names of the repository/repositories and accession number(s) can be found below: https://www.ncbi.nlm.nih.gov/, PRJNA647157.

## Author Contributions

SZ and SS conceived and designed the experiments. SZ, GG, YW, and ZG performed the experiments. SZ, RL, and KW analyzed the data. SZ, RL, ZX, and SS wrote the manuscript. All the authors contributed to the article and approved the submitted version.

## Conflict of Interest

The authors declare that the research was conducted in the absence of any commercial or financial relationships that could be construed as a potential conflict of interest.

## Footnotes

1 https://github.com/wwood/CoverM

2 http://tree.bio.ed.ac.uk/software/figtree

## Supplementary Material

Supplementary Figure 1 Bar plot of the number of DNA read pairs before and after quality control across samples.

Supplementary Figure 2 Diversity of genes encoding enzymes with a role in carbohydrate degradation. The abscissa represents the total numbers of genes encoding GHs, PLs, and CEs in each of the 322 genomes, and the ordinate represents the numbers of CAZyme families encompassing these genes.
