# Supplementary figures and images for: Characterization of Metagenome-Assembled Genomes and Carbohydrate-Degrading Genes in the Gut Microbiota of Tibetan Pig
Source: Front Microbiol. 2020 Dec 23;11:595066. doi: 10.3389/fmicb.2020.595066 (PMC7785962; doi:10.3389/fmicb.2020.595066)

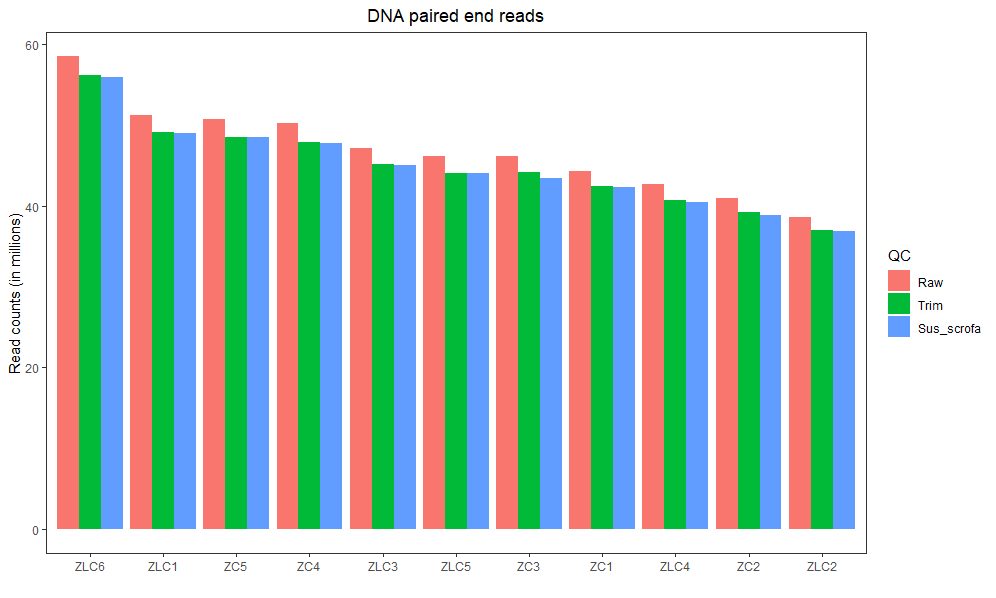

Supplement: Supplementary Figure 1 — Bar plot of the number of DNA read pairs before and after quality control across samples. [file Image_1.TIFF]

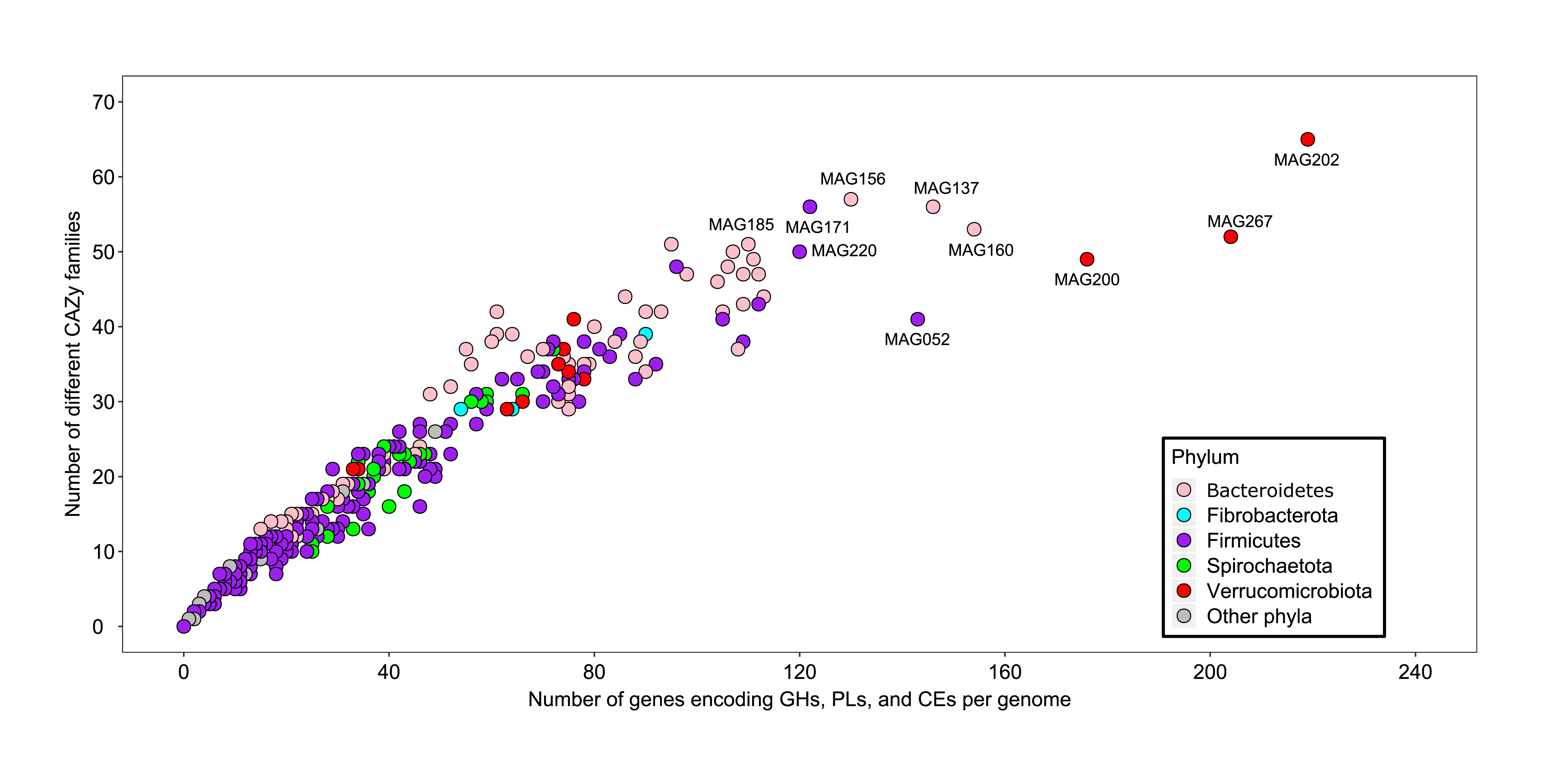

Supplement: Supplementary Figure 2 — Diversity of genes encoding enzymes with a role in carbohydrate degradation. The abscissa represents the total numbers of genes encoding GHs, PLs, and CEs in each of the 322 genomes, and the ordinate represents the numbers of CAZyme families encompassing these genes. [file Image_2.TIF]
